# Planting the seeds for success: A qualitative study exploring primary healthcare providers’ perceptions about medical cannabis

**DOI:** 10.1371/journal.pone.0295858

**Published:** 2024-03-07

**Authors:** Sandi Schuhmacher, Dina Gaid, Lisa D. Bishop, Laura Fleming, Jennifer Donnan

**Affiliations:** School of Pharmacy, Memorial University of Newfoundland, St. John’s, Newfoundland and Labrador, Canada; University of Turin, ITALY

## Abstract

**Background:**

In Canada, cannabis legalization altered the way that the public can access cannabis for medical purposes. However, Canadians still struggle with finding healthcare professionals (HCPs) who are involved in medical cannabis counselling and authorization. This raises questions about the barriers that are causing this breakdown in care. Our study explored the perceptions of primary care providers regarding cannabis in their practice.

**Methods:**

Semi-structured interviews were conducted by Zoom with HCPs in Newfoundland and Labrador (NL) to discuss their experiences with medical and non-medical cannabis in practice. Family physicians and nurse practitioners who were practicing in primary care in NL were included. The interview guide and coding template were developed using the Theoretical Domains Framework (TDF). A thematic analysis across the TDF was then conducted.

**Results:**

Twelve participants with diverse demographic backgrounds and experience levels were interviewed. Five main themes emerged including, knowledge acquisition, internal influences, patient influences, external HCP influences, and systemic influences. The TDF domain resulting in the greatest representation of codes was environmental context and resources.

**Interpretation:**

The findings suggested that HCPs have significant knowledge gaps in authorizing medical cannabis, which limited their practice competence and confidence in this area. Referring patients to cannabis clinics, while enforcing harm-reduction strategies, was an interim option for patients to access cannabis for medical purposes. However, developing practice guidelines and educational resources were suggested as prominent facilitators to promote medical cannabis authorization within the healthcare system.

## Introduction

Cannabis was legalized for medical use in 2001 [1], followed by non-medical use in 2018 [2]. Between 2001 and 2018, patients could access cannabis by getting authorization from a licensed healthcare professional (HCPs) and then purchasing directly from a licensed medical producer [3]. However, the number of registered medical cannabis patients has been decreasing since 2018 with new avenues of access [4]. In line with this, the 2021 Canadian Cannabis Survey reports that of the 14% of Canadians who report using cannabis medically, 78% do so without authorization from an HCP [5].

While the claims for the health benefits of cannabis are vast, the evidence supporting cannabis use for medical indications is quite limited [6]. Pratt et al. [7] conducted a scoping review of systematic reviews on the benefits and harms of medical cannabis; this review concluded inconsistent findings and a lack of rigorous evidence about medical cannabis. As a result of the uncertainty surrounding the health benefits of cannabis, Canadians reported challenges with finding HCPs who were willing to engage in conversations about medical cannabis authorization [3, 8]. Studies have indicated that HCPs do not feel comfortable in this regard [9, 10]. The most common barriers cited were a lack of education and guidelines, the potential for misuse, and a shortage of data on the effects of long-term cannabis use [9–11]. Additionally, the stigma attached to cannabis’ previous illegal status is still prevalent in both the medical world [12] and the greater community [12, 13].

While these barriers have been reported, most of the studies conducted in Canada in this area consist of close-ended surveys rather than qualitative research [9, 10, 12], with a weak representation of Atlantic provinces [9, 14]. Therefore, it is important to explore HCP barriers and facilitators regarding medical cannabis in a more local context to inform policies that will improve shared decision-making. Moreover, adopting a theoretical framework to identify these factors maximizes the likelihood of identifying appropriate behaviour change strategies [15–17].

The Theoretical Domains Framework (TDF) was developed to investigate the determinants of healthcare providers’ behaviour to maximize the uptake of research evidence in healthcare settings [18, 19]. Many research studies have adopted the TDF to explore the barriers and facilitators of healthcare provider behaviours towards implementing evidence-based guidelines [20–22]. This qualitative study used the TDF to determine the perceptions of HCPs on authorizing medical cannabis in their primary care practice in Newfoundland and Labrador (NL).

## Methods

### Research design

Semi-structured telephone interviews were conducted with primary care authorizers (family physicians and nurse practitioners) in NL. This study is reported according to the guidance of the COnsolidated criteria for REporting Qualitative research Checklist [23]. This study was approved by the Health Research Ethics Board at Memorial University of Newfoundland (HREB #2021.199).

### Eligibility criteria

To be eligible, participants had to 1) practice in NL, 2) be registered with either the College of Physicians and Surgeons of NL [CPSNL] or College of Registered Nurses of NL [CRNNL], 3) practice in a primary healthcare setting, 4) be able to prescribe medications, and 5) be able to communicate verbally in English.

### Recruitment strategies

Participants were sought using purposeful recruitment through professional association newsletters (e.g., NL Medical Association, CRNNL), snowball sampling and through our social media channels (Facebook and Twitter). Snowball sampling was particularly helpful to maximize the diversity in our sample with respect to the profession, age, gender identity, years in practice and geographical location. Participants were offered a $100 Amazon gift card.

### Research team

The research team was composed of five researchers, each with a health professional background, with varying levels of research expertise. All researchers were cognizant of their healthcare-related training and professional backgrounds and every effort was made to ensure it did not affect the interviews or data analysis. A neutral perspective was maintained throughout the interviews to avoid any impact on participant responses. Three team members were responsible for leading the interviews (LF, DG, JD) with two being present at each. The interviewers had no prior relationship with the participants.

### Data collection

The semi-structured interview guide (Appendix 1) was adapted from an interview guide used by Elliott et al [14], in combination with the TDF [24]. The interview questions were contextualized to the NL primary healthcare environment. Questions within the guide were modified throughout the study based on what was learned in prior interviews. Field notes were recorded throughout data collection to document interviewer impressions and level of saturation.

### Procedures

The semi-structured interviews were conducted either by phone or videoconference between January 17^th^ and April 30^th^ 2022. Participants provided verbal consent after reviewing the consent and addressing any questions. Interviews lasted between 30 and 80 minutes and were digitally recorded, transcribed verbatim, and de-identified. The transcripts were sent to participants for member checking to ensure accuracy. Interviews were conducted until saturation in the responses was met and we had an adequate representation of different populations.

### Data analysis

Descriptive statistics were used to describe participants’ demographic information. De-identified transcripts were coded using a deductive method guided by TDF [19] and recorded using Excel. Two team members (LF and DG) independently coded the first three transcripts to develop a coding template. The two coders compared their results, resolved discrepancies, and reached a consensus through discussion. The remaining 9 transcripts were coded (SS and DG) and checked for accuracy. The coded data was then further analysed using an indictive thematic analysis approach, where themes that naturally emerged from the data defined. To maximize the analytical rigour, the study team had periodical meetings to discuss and review the coding scheme.

Once all codes were mapped to the TDF domains, the codes were grouped into themes within TDF domains. Each code was categorized as a facilitator, barrier, both, or neutral. An impact score was used to determine which themes and codes had the most impact on healthcare professionals. Codes were ranked following the method used by Islam et al.’s study employing TDF [25]. The impact score was calculated using three criteria: (i) frequency of code, (ii) presence of conflicting beliefs and (iii) evidence of strong beliefs (Table 1). Each criterion was assigned two points for a maximum of six points. Codes which received a score of four or greater were considered most relevant.

**Table 1:** Descriptions of the relevancy criteria.

Criterion	Description
**Frequency**	Subtheme appears in more than half of the interviews (>7).
**Conflicting beliefs**	Participants have opposing views on a subtheme.
**Strong beliefs**	Emphatic language is used or the subtheme is repeated throughout the interview without further prompting.

## Results

We interviewed a total of 12 primary HCPs. Of these eight were physicians and four were nurse practitioners, and represented three of the four NL health authorities (Table 2). The participants had a range of experiences with authorizing cannabis including, not willing to authorize (n = 4), willing to authorize with caveats (n = 5) and currently authorizing cannabis (n = 3). Forty-one unique codes spanning 11 of the 14 TDF domains were identified. Codes were further organized into five emerging themes. Themes included knowledge acquisition, internal influences, patient influences, external HCP influences, and systemic influences. Table 3 outlines each theme with sample quotes organized by TDF domain and code. Table 4 presents the relevancy metric for themes and codes for the TDF domain. The following section presents the major themes in relation to each TDF domain for their relevancy scores.

**Table 2:** Participant characteristics.

Category	Sub-Category	Frequency (N)	Percent
Gender	Woman	9	75%
	Man	2	17%
	Genderqueer	1	8%
Age (years)	30–39	5	42%
	40–49	4	33%
	50–59	1	8%
	60–69	2	17%
Profession	Nurse Practitioner	4	33%
	Physician	8	67%
Health authority	Eastern	9	75%
	Central	1	8%
	Labrador-Grenfell	2	17%
Experience (years)	1–9	2	17%
	10–19	7	58%
	20–29	1	8%
	30–39	2	17%

**Table 3:** Coding guide separated into 5 overarching themes with associated Theoretical Domains Framework domains and representative quotes.

**Theme**	**Knowledge Acquisition**				
**TDF Domains**	**Code**	**Example Quotes**	**Barrier**	**Facilitator**	**Neutral**
Environmental context and resources	Availability of quality evidence	"We don’t have the kind of really good trials to let us know how, when at what dose, frequency and with what, with what potential side effects." (P8)	✓		
		“So maybe it would be nice to have that a little bit more robust evidence in a clinical trial, say, for cannabis to be able to guide us a little bit more to say who would do better under what circumstances” (NP2)		✓	
	Evidence evolving	"I guess it reminds us to be ever so keen on keeping up on, and you know, current, what the current research shows, so I am sure as the years go by there will certainly be. . . lots more studies" (NP1)		✓	
	Availability of resources	"With cannabis, you feel kind of isolated, we’ll say on that topic because there’s not many, in my opinion, there’s that many resources or a team approach to it, we’ll say outside of a referral to the Cannabo Clinic." (NP3)	✓		
		“the College of Family Physicians of Canada puts out a monthly journal which often have current things in it, and I’m sure they’ve had stuff on cannabis” (P6)		✓	
	Availability of continuing education	"And I think I’ve never been offered any, to be honest in terms of, or any opportunities like I haven’t seen any in my memory that there was an opportunity to get any extra training in it” (P10)	✓		
		“I did listen to a webinar that and it was mainly about like, you know, prescribing different forms of cannabis, but it maybe wasn’t as beginner as I need it to be” (NP4)		✓	
	Accessibility of continuing education	"I know the physicians that I meet in the hallway, I don’t say they ever have the opportunity during the day, umm, during regular hours, I’ll say, our patient care hours, to avail of some of these things” (NP1)	✓		
		“So, you know, that’s certainly, sometimes all it takes is, sometimes I’ll get emails and it will be an educational session thing and I will be like, oh, I don’t know a lot about that or something or I never thought that it’s something I should look into but then once I, so I’ll sign on to things like that, I realize as well how much I need to learn about area” (NP1)		✓	
	Quality of continuing education	“There’s, there’s lots of stuff out there. The problem is not finding a CME. The problem is finding a CME that’s worth an hour of your time” (P8)	✓		
	HCP school curriculum/ formal education	“when I went through uh, nurse practitioner school, now that was not very long ago, I graduated in 2019, it wasn’t really something that we did to be honest, we, I don’t really recall touching on it a whole lot at all” (NP1)	✓		
Knowledge	Knowledge of information sources	"I use a lot of databases here, up to date, those types of things, you know, I use them on a daily basis" (NP1)		✓	
	Indication for cannabis use	“With respect to the medical part, I know there’s lots of controversy about who, you know, the indications and who should be treated for the indications it can be treated for versus the actual evidence” (P9)	✓		
		"So, I do a lot of palliative care. So, I’ve had patients use medical cannabis for pain control and for nausea, for sleep. And I’ve also had patients who use medical cannabis for, say, mental health like PTSD” (NP4)		✓	
**Theme**	**Internal Influences**				
**TDF Domains**	**Code**	**Example Quotes**	**Barrier**	**Facilitator**	**Neutral**
Knowledge	Current level of knowledge	"No, I guess…, my own barrier would be lack of, lack of knowledge, you what I mean, that would be my biggest personal barrier" (NP1)	✓		
		“I’m usually fairly keep it fairly general to say that, you know, there has been some benefit to medical cannabis in an anti-inflammatory perspective so again 90 percent of the time people are coming from chronic pain” (NP2)		✓	
Skills	Competence	"Yeah, so with CBD, which is where I almost always start, I think it takes about a month to assess. So, I book a six week follow up to give them time to order and try out the product. And I have a. . . most common titration schedule. . .and then I adapt it to the to the particular person’s life situation” (P11)		✓	
Social/ professional role and identity	Professional identity	“I mean, it wasn’t until 2019 that nurse practitioners were allowed to prescribe cannabis.” (NP4)	✓		
	Professional role	"I want to do whatever I can possibly do for my patients within my scope, within my comfort level, within what’s reasonable for medicine." (P10)		✓	
	Professional boundaries	"I certainly offer it to say, you know, we have a cannabis clinic, I can’t prescribe cannabis because I don’t have that licensure. However, if it’s something you’d be interested in discussing, you know, we have this service” (NP2)			✓
Beliefs about capabilities	Perceived competence/ comfort	“I don’t have, I don’t have the kind of science that I would like to have to be able to prescribe this with confidence” (P8)	✓		
		"I feel comfortable. But I don’t know if I have as much knowledge as I probably do on other things, I think because the I guess the evidence is still emerging and still new” (P5)		✓	
	Professional confidence/ willingness	"I can certainly see myself prescribing… I’m a believer that, you know, certainly when you’re in primary health care, within reason and within your own comfort, you know, ultimately you should try your best to offer whatever your patients would need” (NP2)		✓	
	Attitude towards cannabis	“I think I probably have a bit of an unconscious bias against it” (P6)	✓		
		“I am certainly am open to and welcome anything that may help my patients” (NP1)		✓	
Optimism	Optimism	"I am really hopeful now, like I said, that some of [the barriers] will soon start to change, by soon, that might be 2030 unfortunately, but you know, hopefully before that." (NP1)		✓	
	Place in therapy (belief in use)	“that’s probably the instance that people inquire about, if not or that I would bring up about the most would be chronic pain. But we know it’s not first line, so I certainly don’t bring it up, I think it’s third line if you look at the guidelines for non-cancer pain” (NP2)		✓	
Goals	Harm reduction philosophy	"I tend to operate from a harm reduction philosophy that if people are going to do things that maybe I wouldn’t necessarily recommend, I still want to make sure they’re doing it in a safe way as reasonable and support them as much as I can and do something as safely as possible” (NP2)		✓	
Environmental context and resources	Suggested facilitators	"I would love to see it regulated to the same extent as other medications. Be able to track it on… [the pharmacy electronic record], as they do other medications” (P9)		✓	
	Past experiences	“I do have some people who umm you know, use the CBD oil for example, regularly, they identify that it certainly does help them. With the sleep portion, that they experience sleep disruption and things like that” (NP1)		✓	
		“In her case, it was, yeah, let’s give it a try and see if it makes any difference. In her case, it really didn’t. It helped her appetite, which I laugh about because her family found that beneficial, but she eventually stopped it. She didn’t find that it helped her.” (P7)	✓		
Intentions	Stage of change	"Yeah, until I am more satisfied with the process, that there are better guidelines about dosing for the oils…I’ve decided not to prescribe it." (P9)	✓		
		"I wouldn’t [authorize cannabis] right now. I’m not saying I won’t down the road, but based on my comfort level with it right now, I wouldn’t because of my education and my prescribing experience with it." (NP3)	✓		
**Theme**	**Patient Influences**				
**TDF Domains**	**Code**	**Example Quotes**	**Barrier**	**Facilitator**	**Neutral**
Reinforcement	Harm to patients/ misuse	"I’ve seen patients in drug use psychosis, from you know, marijuana use and things like that." (NP1)	✓		
Goals, reinforcement	Benefit to patients	"I have seen people have just done so much better on cannabis than they’ve done with anything else." (NP2)		✓	
Environmental context and resources, reinforcement	Patient openness	“one specifically just mentally not ready to even consider if it is something that she wants to try” (P7)	✓		
		"If you show a willingness to talk about it, you’d be surprised how willing patients are to talk about it." (P8)		✓	
	Patient knowledge	"For some people, that’s not the case, you know, they, they’ve been doing this for quite some time and you know, nobody’ has ever told them that maybe, you know, there are potential risks" (NP1)	✓		
		“I would say about 25 percent of people have what I’ll call a reasonable understanding of cannabis, in that’s what they know is what I’ll say factual information and not just information” (NP2)		✓	
	Patient/ caregiver autonomy	“they’ve tried some various things on the market that they have certainly been able to um to purchase themselves or obtain themselves” (NP1)		✓	
	Patient access point	"Because it’s been so illegal for so long, there’s still, most of the stuff that young people are smoking is not from Tweed. It’s too expensive. They’re mostly getting it from the street" (P5)	✓		
		“…they seem to know more about the strains, and I don’t even know about any of that. So, there’s definitely online companies that do that for some people. I’ve got a couple of patients that are really into it, and they go through these companies online and figure out which strain to use” (P5)		✓	
	Patient barrier	“The mines have zero tolerance policy for any sort of drug, marijuana, anything. So, that has been a barrier for some people” (NP1)	✓		
	Demand for information	"To be honest, when it first became legal was, I guess, a couple of years ago, I got a lot more questions about it, about people wondering about it as options. But I find now that it’s been legalized for a while, I don’t get as many questions anymore." (P5)		✓	
	Demand for authorization	“Well, you know, when it was first legalized, I had a lot of patients asking for prescriptions, and I really didn’t, I felt kind of blindsided” (P9)	✓		
		“they had probably tried it on, they probably bought it on the street and tried it. That’s why they wanted it authorized because they knew that it worked” (P8)		✓	
	Culture shift	"certainly since it’s been legalized, there’s a lot more, I’ve had a lot more questions about it” (P6)		✓	
Social influences	Social norms	"Yeah, I think it, uh, it’s our culture now, I mean, for the most part, it’s, it’s a normal part of our culture I guess” (NP1)		✓	
	Patient influence on decisions	“And I find the people who ask for the authorizations generally are looking for the oils.” (P10)		✓	
	Patient beliefs	“And there’s a scattered one or two that I have my clinic who are severely depressed, and the cannabis is definitely adding to it and they’re using it all the time and think there’s no reason for them to, to stop and they think it’s helping, but it really isn’t right.” (P5)	✓		
		"They love the idea of you know, kicking it to big pharma. You know that they’re not taking your prescription medication, that they’re taking something natural. So, I do wonder sometimes if there’s a little bit of a confirmation bias there." (NP2)		✓	
**Theme**	**External HCP Influences**				
**TDF Domains**	**Code**	**Example Quotes**	**Barrier**	**Facilitator**	**Neutral**
Social influences	External practitioner knowledge/ competence	"I do find like when I send patients to the cannabis clinic, they do very, very thorough assessments, they do very thorough documentation" (NP2)		✓	
	External practitioner views	"There is still a certain stigma when you’re talking about it among colleagues, but not among patients" (P8)	✓		
Environmental context and resources	Cannabis specialists available	“But it is a clinic here in town where I’ve referred people to, and it’s more the pros and cons are reviewed there, where they just make a more informed decision than what they do with me.” (P7)		✓	
**Theme**	**Systemic Influences**				
**TDF Domains**	**Code**	**Example Quotes**	**Barrier**	**Facilitator**	**Neutral**
Environmental context and resources	HCP workplace policy	"No, we don’t we don’t have any policies, we don’t really authorize cannabis."(P5)			✓
	Systemic barriers	"So, yeah, the authorization piece…is more to do with the bureaucracy, less to do with, with the concern around dose and frequency" (P8)	✓		

**Table 4:** Relevancy matrix for the themes and the codes in relation to the Theoretical Domains Framework.

TDF Domains	Themes				
	Knowledge Acquisition	Internal Influences	Patient Influences	External HCP Influences	Systemic Influences
**Environmental context and resources**	Availability of quality evidence	Suggested facilitators	Patient openness	Cannabis specialists available	HCP workplace policy
	Evidence evolving	Past experiences	Patient knowledge		Systemic barriers
	Availability of resources		Patient/caregiver autonomy		
	Availability of continuing education		Patient access point		
	Accessibility of continuing education		Patient barrier		
	Quality of continuing education		Demand for information		
	HCP school curriculum/formal education		Demand for authorization		
			Culture shift		
**Knowledge**	Knowledge of information sources	Current level of knowledge			
	Indication for cannabis use				
**Skills**		Competence			
**Social/professional role and identity**		Professional identity			
		Professional role			
		Professional boundaries			
**Beliefs about capabilities**		Perceived competence/comfort			
		Professional confidence/willingness			
		Attitude towards cannabis			
**Optimism**		Optimism			
		Place in therapy (belief in use)			
**Goals**		Harm reduction philosophy	Benefit to patients		
**Intentions**		Stage of change			
**Reinforcement**			Harm to patients/ misuse		
			Benefit to patients		
**Social influences**			Social norms	External practitioner knowledge/competence	
			Patient influence on decisions	External practitioner views	
			Patient beliefs		
**Legend**					
	IS = 6	IS = 4	IS = 2	IS = 0	

IS: Impact Score; TDF: Theoretical Domains Framework

### Knowledge acquisition

The most prominent theme was knowledge acquisition and codes related to this theme appeared in two TDF domains, environmental context and resources, and knowledge. Participants emphasized the challenges to the availability and access to evidence, resources, and continuing education. Six of the nine codes mapped to this theme had an impact score of six, suggesting a high level of relevance.

### Internal influences

Participants commented on many internal influences that impacted their role in supporting patients’ cannabis decisions. These included concepts related to professional competence, roles, goals and identities, personal attitude towards cannabis and their level of optimism for cannabis playing a role in patient care, and their intentions when it comes to cannabis authorization. While most participants did not feel that they possessed the necessary competence to adequately advise patients on cannabis use, most were optimistic regarding it having a place in therapy for some people. Codes related to this theme appeared in eight TDF domains of which half (7 out of 14 codes) had an impact score of six.

### Patient influences

Clinicians also discussed how their patients played a role in shaping their positions on medical cannabis use. Their personal experiences in caring for patients who have had either beneficial clinical outcomes or serious adverse reactions to cannabis can influence how they care for and advise other patients. Barriers faced by patients also had an impact on the clinician’s willingness to authorize. Few patients have insurance that covers cannabis, and many employers have strict policies that prohibit cannabis. Generally speaking, clinicians noted that patients have become more open to cannabis as an alternative therapy and often come to their appointments with some prior knowledge and formed opinions. Since legalization, they have noticed a shift, with fewer requests for authorization which were speculated to be related to the ease of access through the non-medical market. Codes related to this theme appeared in four TDF domains, and two of the 14 codes had an impact score of six.

### External healthcare professional influences

Three codes were mapped to the theme of external HCP influence, and each of those codes had a moderate or high impact score. In particular, almost every participant discussed the availability of a specialized cannabis clinic where they could refer patients. This removed many of the barriers that individual clinicians faced concerning professional knowledge and confidence. Some participants discussed prevailing stigma in the medical community, and how that may influence their colleagues.

### Systemic influences

The final theme was systemic influences. This included two codes, one of which had an impact factor of six. There was a distinction between professions in this theme with nurse practitioners citing regulations as their greatest systemic barrier and primary physicians being deterred by the authorization process. While many HCPs agreed with systemic barriers being a concern, none were aware of any policies present in their workplace preventing them from authorizing medical cannabis.

### Interpretation

Our study aimed to determine HCP perceptions on their ability and comfort in supporting their patients with decisions regarding medical cannabis in their primary care practice. Our findings suggested that the most prominent concerns were related to the availability of evidence, resources, and continuing education; this also supports the finding that HCPs felt that they lacked confidence and competence in this area.

Prominent factors reported by our participants included the lack of knowledge related to the cannabis authorization process including dosing, routes of administration, process requirements and ease, and scope of professional practice. Similar gaps have been reported among physicians in the USA [26, 27] and Canada [9, 28] regarding low levels of medical cannabis knowledge. Aligning with previous studies [9], another prominent resource-related barrier disclosed by our participants was the lack of available evidence related to clinical practice treatment guidelines and clinical research demonstrating cannabis safety authorization [28]. Our study additionally highlighted the importance of health system-related factors including the clarity of cannabis authorization policy as our respondent’s denied knowledge of any specific policies in their workplace that prevent them from authorizing cannabis.

This knowledge acquisition gap can negatively impact HCPs’ attitude, confidence and competence to authorize cannabis. Our findings suggested that HCPs have overall receptive attitudes towards cannabis as a therapeutic option but do not feel equipped to support patient decisions, authorize or counsel on it due to a lack of knowledge and resources. Previous research also indicated that the majority of HCPs considered themselves unprepared to advise their patients about medical cannabis [29]. However, the comfort to authorize medical cannabis was positively correlated with more experience in that area [30, 31]; consistent with our findings.

Despite reported gaps in knowledge, lack of confidence, and unawareness of organizational policies, all participating HCPs were open to speaking to their patients about medical cannabis and offering guidance. Patients have also become more open to discussing medical cannabis as a treatment option, as it is now widely accessible legally for non-medical use. Previous research has been divided on the ease with which patients can discuss medical cannabis with their HCPs [32]. While some studies indicate that patients face challenges discussing medical cannabis with HCPs, other literature reports some patients consider HCPs to be their main source of information about cannabis [33] and are more willing to consider its use when suggested by their trusted HCP [34]. This evidence highlights the value patients place on their relationship with their HCPs and our findings support the need to strengthen training and resources available to clinicians to fill this care gap.

Moreover, some participants reported the disconnection between medical cannabis and other drug products as cannabis is often not documented in the electronic health record and therefore does not go through the same safety or drug interaction checks. The lack of pharmacist involvement raises safety considerations since licensed cannabis retailers are legally not permitted to provide any health-related information [35–37]. Thus, the role of HCPs extends beyond providing cannabis consultation or authorization. Research has shown that many individuals self-medicate with cannabis and participants in our study expressed their role in supporting a harm reduction approach, which helps HCPs to guide their patients toward safe cannabis use [38, 39], by recommending access through regulated sources, discussing usage patterns to avoid cannabis misuse and suggesting routes of administration other than smoking.

This study provides several suggestions to regulate the process of medical cannabis authorization. First, the development of regulatory guidance for medical cannabis authorization informed by clinical research is highly needed to minimize the knowledge gap. Researchers have endeavored to develop clinical practice guidelines for medical cannabinoid use. Allan et al. [40] have recommended against the use of medical cannabis for acute pain, rheumatologic pain, neuropathic pain, cancer pain, nausea and vomiting, and spasticity. While, Bell et al. [41] have recently reported moderate benefits of medical cannabis in managing chronic pain and other comorbidities including sleep problems, anxiety, appetite suppression, and managing pain associated with arthritis, HIV, multiple sclerosis, and fibromyalgia. As rigorous/consistent guidance is still lacking, clinical research should be prioritized to guide the development of medical cannabis clinical practice guidelines to inform HCPs in providing effective care for patients [9]. Second, offering cannabis-related education programs for the HCPs would be low effort and high reward; these programs have been highlighted as a critical need to address the knowledge gaps among HCPs [9, 26, 27, 29, 42–44]. Lastly, education for students training to be nurse practitioners and physicians could address some of the growing demand of education on medical cannabis for therapeutic purposes by incorporating learning within the curriculum [27–29, 43, 45]. In general, HCPs in Canada and around the world have low exposure to cannabis-related topics in their education curricula [10, 45–47].

### Limitations

The research was conducted with a select number of providers in one province, so the findings may not be transferable to other jurisdictions or all practice settings. However, our research provides a general insight into the common factors that can be considered in primary care settings. We only explored the perspectives of family physicians and nurse practitioners; exploring the perspectives of other HCPs and patients would provide different insights into the barriers and facilitators around medical cannabis use and access.

### Conclusion

HCPs have knowledge gaps in authorizing cannabis for their patients, which limited their practice competence and confidence in this area. However, providers were open to discussing cannabis as an option with their patients. Referring patients to cannabis clinics, while enforcing harm-reduction strategies, was an alternative option for patients to access cannabis for medical purposes. However, developing practice guidelines and educational resources were suggested as ways to support primary care providers medical cannabis authorization within the healthcare system.
